# Cholinergic Regulation of Airway Inflammation and Remodelling

**DOI:** 10.1155/2012/681258

**Published:** 2012-01-16

**Authors:** Saeed Kolahian, Reinoud Gosens

**Affiliations:** ^1^Department of Basic Sciences, Faculty of Veterinary Medicine, University of Tabriz, Iran; ^2^Department of Molecular Pharmacology, University of Groningen, Antonius Deusinglaan 1, 9713 AV Groningen, The Netherlands

## Abstract

Acetylcholine is the predominant parasympathetic neurotransmitter in the airways that regulates bronchoconstriction and mucus secretion. Recent findings suggest that acetylcholine regulates additional functions in the airways, including inflammation and remodelling during inflammatory airway diseases. Moreover, it has become apparent that acetylcholine is synthesized by nonneuronal cells and tissues, including inflammatory cells and structural cells. In this paper, we will discuss the regulatory role of acetylcholine in inflammation and remodelling in which we will focus on the role of the airway smooth muscle cell as a target cell for acetylcholine that modulates inflammation and remodelling during respiratory diseases such as asthma and COPD.

## 1. Introduction

Acetylcholine is classically viewed as a neurotransmitter that regulates cognitive and behavioural functions in the brain, autonomous ganglionic transmission, and parasympathetic postganglionic transmission. In the respiratory tract, acetylcholine is the predominant parasympathetic neurotransmitter and its role in the regulation of bronchomotor tone and mucus secretion from airway submucosal glands is well established [1]. More recent findings suggest that acetylcholine regulates additional functions in the respiratory tract, including inflammation and remodelling during inflammatory lung diseases [2–4]. Moreover, it has become apparent that acetylcholine is synthesized by nonneuronal cells and tissues, particularly inflammatory cells and the airway epithelium [5–7]. These cells also express receptors for acetylcholine, including muscarinic receptors and nicotinic receptors that modulate inflammatory responses [2, 6]. Collectively, these findings have questioned the traditional view on the physiological and pathophysiological role of acetylcholine, which has opened up new possibilities for therapeutic targeting of the pulmonary cholinergic system. In this paper, we will discuss these recent findings in which we will focus on the role of the airway smooth muscle cell as a target for acetylcholine in inflammation and remodelling during respiratory diseases such as asthma and COPD.

## 2. The Origin of Acetylcholine

Acetylcholine is biosynthesized from choline and acetyl-CoA by choline acetyltransferase (ChAT) or carnitine acetyltransferase (CarAT) by several cell types in the respiratory tract [6]. Airway neurons and airway epithelial cells express ChAT and have been demonstrated by HPLC detection to release acetylcholine [5]. The release of acetylcholine from other nonneuronal tissues in the respiratory tract is suggested by the fact that also macrophages, mast cells, fibroblasts, smooth muscle cells, lymphocytes, and granulocytes express ChAT immunoreactivity [6]; however the release of acetylcholine from these cells and tissues has not yet been measured directly in the respiratory tract. Acetylcholine exerts its functions either via muscarinic receptors, a class of G-protein-coupled receptor subtypes, or via nicotinic receptors, a class of ligand-gated cation channels [8]. Most structural cells and inflammatory cells that are present in the respiratory system, including smooth muscle cells, fibroblasts, epithelial cells, mast cells, granulocytes, lymphocytes, and macrophages, express muscarinic and/or nicotinic receptors [2, 6]. For a detailed overview of individual receptor subtypes and subunits expressed by these cells, we refer to a recent excellent overview by Wessler and Kirkpatrick [6]. The expression of muscarinic and nicotinic receptors, the expression of synthesizing enzymes such as ChAT, and the direct measurement by HPLC detection of acetylcholine release from nonneuronal tissues and cell cultures are solid evidence for the existence of a nonneuronal cholinergic system in addition to the more established neuronal cholinergic system in the airways.

The processing of acetylcholine by nonneuronal cells and tissues is not yet described in full although, for airway epithelial cells, secretory mechanisms have been described. Airway epithelial cells express the high affinity choline transporter (CHT1) that is involved in choline uptake as well as the organic cation transporter (OCT) subtypes 1 and 2, which play a dominant role in the release of acetylcholine by airway epithelial cells [9, 10]. Furthermore, the expression of the vesicular acetylcholine transporter (VAChT) by epithelial cells has been reported suggesting that storage of acetylcholine in vesicles and release via the fusion of these vesicles with the plasma membrane, as occuring in neurons, may represent an additional mechanism for acetylcholine release by nonneuronal cell types [9, 10].

The breakdown of acetylcholine into acetic acid and choline is catalysed by acetylcholinesterase (AChE) and butyrylcholinesterase (BuChE), also known as pseudocholinesterase. The functional expression of AChE by airway epithelial cells is evidenced by observations that acetylcholine concentrations in cell supernatants of airway epithelial cell cultures were enhanced by the pharmacological inhibitor of AChE, neostigmine [5]. Collectively, the above-mentioned observations indicate that both neurons and nonneuronal cells and tissues in the respiratory system express and release acetylcholine. The functional role of nonneuronal acetylcholine on the airway smooth muscle includes bronchoconstriction [11, 12]. Additionally, acetylcholine may modulate airway hyperresponsiveness and remodelling, including the regulation of airway smooth muscle growth and the regulation of airway inflammation that promotes hyperresponsivness and remodelling. This role for acetylcholine will be discussed in the following sections.

## 3. The Muscarinic Receptor: Acetylcholine as a Proinflammatory and Remodelling Mediator

Muscarinic receptors are expressed by most structural cells in the airway wall, including the airway smooth muscle and by inflammatory cells that are involved in the pathogenesis of obstructive airway diseases [2]. Muscarinic receptors appear to play a proinflammatory role on these cells, suggesting that inhibition of muscarinic receptor function may have anti-inflammatory effects in these diseases. Increased expression of muscarinic M_1_ and M_3_ receptors on airway structural cells and sputum cells of COPD patients has been reported [13, 14]. Likewise, reduced expression of the autoinhibitory M_2_ receptor on airway neurons in asthma has been reported [1]. Both effects could contribute to enhanced acetylcholine release and function in these diseases. The proinflammatory role of acetylcholine via muscarinic receptors is discussed below.

### 3.1. Direct Effects of Acetylcholine on Airway Smooth Muscle

The airway smooth muscle expresses muscarinic M_2_ and M_3_ receptors roughly in a 4 : 1 ratio [15]. The muscarinic M_3_ receptor represents a primary target of acetylcholine in the airways, involved in the regulation of bronchoconstriction [15–18]. In addition, muscarinic receptors regulate proliferative and proinflammatory functions of the airway smooth muscle. It was observed that coadministration of muscarinic agonists with epidermal growth factor (EGF) in human airway smooth muscle cells induces a synergistic proliferative stimulus. This effect was associated with sustained activation of p70 S6 kinase [19, 20], an effect mediated by G_q_-derived G_*βγ*_ subunits that activate phosphatidylinositol-3-kinase (PI3K) in concert with the EGF receptor [19, 21]. In line with these findings, muscarinic receptor agonists induce an increase in proliferation of airway smooth muscle cells in combination with platelet-derived growth factor (PDGF) [22], which is mediated by G_q_-protein-coupled muscarinic M_3_ receptors and appears to involve a synergistic inhibitory phosphorylation of glycogen synthase kinase-3 (GSK-3) [23]. GSK-3 is a multitasking enzyme that regulates multiple signalling proteins and transcription factors involved in contractile protein expression and cell proliferation of airway smooth muscle [23–26].

 Muscarinic-receptor-induced airway remodelling could also involve mechanical regulation as airway smooth muscle constriction results in airway epithelial cell compression and subsequent activation of EGFR phosphorylation in the airway epithelium [27]. Indeed, a recent clinical trial demonstrates that repeated methacholine inhalations cause airway remodelling in the absence of inflammation, characterized by collagen deposition and increased TGF-*β*1 expression [28]. It is not yet clear whether such effects could also directly regulate remodelling of airway smooth muscle; however, mechanical strain of airway smooth muscle regulates cell proliferation and contractile protein expression [29–31], an effect enhanced in the presence of carbachol [32]. Clearly, this hypothesis needs to be followed up in future studies.

Muscarinic receptors on airway smooth muscle cells could also play a profound role in regulating the immunomodulatory function of airway smooth muscle [33, 34]. Cholinergic stimulation with the muscarinic receptor agonist carbachol augments inflammatory gene expression in bovine tracheal smooth muscle in combination with cyclic stretch, which induces a synergistic increase in the expression of IL-6, IL-8, cyclo-oxygenase (COX) 1 and 2, and urokinase-type plasminogen activator (PLAU) [35]. It was recently demonstrated that the activation of muscarinic receptors also interacts with several cytokines and growth factors that play an important role in the pathogenesis of asthma and COPD, in particular with TNF-*α*, PDGF-AB and cigarette smoke to enhance their inflammatory response in airway smooth muscle cells [36]. Thus, muscarinic M_3_ receptor stimulation of airway smooth muscle with methacholine induces IL-6 and IL-8 production and augments the release of these cytokines induced by cigarette smoke extract [36]. Our unpublished data show that this effect is dependent on downstream signalling to PKC, which activates the I*κ*B*α*/NF-*κ*B and MEK/ERK1/2 pathways [37]. This indicates that acetylcholine may also play an important role in the immunomodulatory processes driven by human airway smooth muscle.

**Figure 1:**
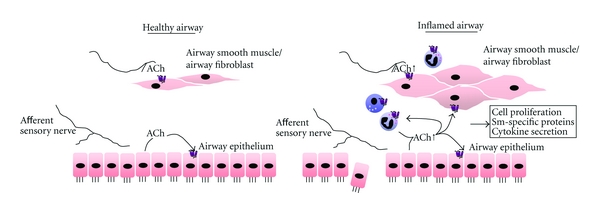
Muscarinic receptor regulation of airway inflammation and remodelling. In healthy airways, acetylcholine release from neuronal and nonneuronal origins release are limited. However, in response to environmental factors such as allergen or smoke, acetylcholine release is enhanced, which cooperates with proinflammatory cytokines and growth factors to induce airway smooth muscle and fibroblast cell responses including cell proliferation, smooth-muscle-specific protein expression, and the synthesis of chemokines and cytokines. As such, acetylcholine by acting on muscarinic receptors may contribute to both acute and chronic aspects of obstructive airways disease. Nicotinic receptors are expressed by airway structural cells and inhibit inflammatory cell activation; however, their role in regulating airway remodelling is largely unknown.

 The functional importance of these *in vitro* findings is illustrated by our *in vivo* studies that indicate a protective role for tiotropium bromide, a long-acting muscarinic antagonist, in the progression of airway smooth muscle remodelling. Thus, guinea pigs challenged with allergen for 12 consecutive weeks developed increased airway smooth muscle mass, increased contractile protein expression, and increased airway smooth muscle contractility, which were partially to fully prevented by treatment with tiotropium bromide [38]. In part, these inhibitory effects may have been due to the anti-inflammatory properties of tiotropium as airway eosinophilia was almost completely reduced by treatment with this compound [39]. Mucus gland remodelling and MUC5A/C hypersecretion were also prevented [39]. These results indicate that acetylcholine plays an essential role in remodelling of the airway smooth muscle (Figure 1). These effects may be direct, as suggested by the *in vitro* studies mentioned above, or indirect, as illustrated below.

### 3.2. Additional Effects of Acetylcholine on Airway Remodelling

Airway smooth muscle cells are embedded in the airway wall, and bidirectional communication between the muscle layer and the cell types and matrix protein structures that surround the muscle bundle is key to the development of abnormalities in airway smooth muscle phenotype and function in obstructive airways disease [40]. Fibroblasts are key effector cells in the production of extracellular matrix proteins that surround the airway smooth muscle bundle in the adventitia and submucosa of the airway wall [41]. Fibroblasts express functional muscarinic M_2_ and M_3_ receptors (predominantly M_2_ receptors with relatively fewer M_3_ receptors) [42]. *In vitro*, the muscarinic agonists carbachol and oxotremorine cause an increase in (^3^H)-thymidine incorporation (as a measure of cell proliferation) in human lung fibroblast cell lines and primary fibroblasts. This effect is mediated by the M_2_ receptor and regulated by the MEK/ERK1/2 pathway [42, 43]. Tiotropium, a long-acting muscarinic antagonist, concentration-dependently inhibited ACh-induced proliferation of primary human fibroblast isolated from biopsies of lung fibrosis patients and myofibroblasts derived from these cells [44]. Furthermore, it was found that muscarinic agonists stimulate the incorporation of ^3^H-proline into cellular proteins (as a measure of collagen synthesis) in human lung fibroblast cell lines and primary fibroblasts [45]. Also, tiotropium bromide inhibits collagen expression in the lung and small airways in guinea pigs repeatedly exposed to LPS [46]. Collectively, these studies support a role for acetylcholine in regulating fibroblast cell responses associated with remodelling.

A proinflammatory role of acetylcholine in fibroblasts was recently questioned by a study showing that the release of chemotactic mediators was not induced in fibroblasts incubated with acetylcholine because of a relative lack of M_3_ receptor expression in these cells [47]. On the other hand, primary lung fibroblast cultures from surgical specimens of COPD patients treated with acetylcholine showed enhanced IL-8 and matrix metalloproteinase-2 release. This effect was mediated by muscarinic M_3_ receptors [14], and tiotropium has an attenuating effect on metalloproteinase-2 production from lung fibroblasts induced by inflammatory stimulation [48]. It is possible that the enhanced expression of muscarinic receptors by fibroblasts of COPD patients explains the discrepancy between these two studies as Profita et al. [14] showed that muscarinic M_1_ and M_3_ receptor as well as ChAT expressions were increased in fibroblasts from COPD patients. Although the quantification of muscarinic receptor expression using antibodies should be approached with care [49], functional differences between healthy controls and COPD patients were also observed. In this study, acetylcholine induced a significant increase in the activation of the ERK1/2 and NF*κ*B pathways in fibroblasts of patients with COPD and promoted cell proliferation to a greater extent than observed in fibroblasts of healthy controls [14]. These findings clearly indicate the function of fibroblasts in remodelling processes that occur in chronic inflammatory airway diseases but the proinflammatory role of lung fibroblasts in response to acetylcholine remains to be studied in further detail.

The airway epithelium is key to the development of airway inflammation and remodelling as it presents the first barrier to inhaled particles and allergens and regulates the secretion of proinflammatory cytokines. Epithelial damage during allergic airway inflammation plays a key role in asthma and exposes sensory nerve endings in the submucosa to the airway lumen, which promotes reflex mechanisms leading to enhanced vagal release of acetylcholine [40]. Moreover, the airway epithelium is predominant in its expression of ChAT and may present a direct source of nonneuronal acetylcholine [5]. Acetylcholine is a proliferative stimulus for human bronchial epithelial cells in culture in part by activation of muscarinic M_1_ receptors [50, 51]. Acetylcholine also increased eosinophil, monocyte, and neutrophil chemotactic activity by bronchial epithelial cells [52, 53]. This effect probably involves muscarinic M_1_ receptors that induce leukotriene B_4_ release from epithelial cells, which in turn stimulates eosinophil, neutrophil, and monocyte chemotactic activities [52, 53]. Muscarinic receptor agonists also induced the release of prostanoids from airway epithelial cells. Thus, muscarinic M_3_ receptors promote the activation of phospholipase A2, which stimulates the release of PGE_2_ from isolated tracheae, but only in preparations with an intact epithelial layer [54]. In addition, a recent investigation in human bronchial epithelial cells showed that acetylcholine induces the production of IL-8, involving PKC, ERK1/2, and NF*κ*B pathway activation via muscarinic receptors [55].

Collectively, these findings indicate that acetylcholine, derived from the vagal nerve and from nonneuronal origins such as the airway epithelium, may induce cell responses associated with airway wall remodelling and trigger proinflammatory cytokine release by structural cells of the airway wall, including airway epithelial cells, airway fibroblasts, and the airway smooth muscle itself. These mechanisms may promote airway inflammation and remodelling, including airway smooth muscle thickening.

### 3.3. Indirect Effects: Acetylcholine as a Proinflammatory Mediator

Airway inflammation in asthma and COPD likely plays an important role in the development of airway hyperresponsiveness and in the development of structural changes in the airway wall including increased airway smooth muscle mass. Inflammatory cells secrete cytokines and growth factors that induce a proliferative stimulus in airway smooth muscle cells (e.g., EGF, PDGF, and TGF-*β*) that may be amplified by the actions of acetylcholine as outlined above [56]. Moreover, acetylcholine, either from neuronal or nonneuronal origin, may regulate inflammatory cell responses in these diseases that explain the beneficial effects of anticholinergics on airway smooth muscle thickening [2].

The anticholinergic agent tiotropium bromide prevented allergen-induced airway eosinophilia in guinea pigs, indicating that muscarinic receptor signalling supports airway eosinophilia [39]. It has been demonstrated that muscarinic M_3_ and M_4_ receptors are expressed in human and guinea pig eosinophils; human eosinophils also appear to express the muscarinic M_5_ receptor subtype [57]. However, Verbout et al. found an inhibitory effect of these muscarinic receptors on eosinophil activation [58]. Atropine, a nonselective muscarinic receptor antagonist, significantly potentiated antigen-induced eosinophil activation and airway hyperreactivity by increasing major basic protein deposition in the airways [58]. The inhibitory effect of muscarinic recepors on eosinophil activation in antigen-challenged animals is mediated by their suppressive effect on excitatory nerve growth factor (NGF) pathway [59]. The effect of muscarinic receptors on airway structural cells (epithelial cells, fibroblasts, airway smooth muscle cells) as outlined above may account for this discrepancy as proinflammatory cytokine production by these cells, including the release of eosinophil chemotactic activity, is enhanced by muscarinic receptor stimulation.

Muscarinic receptors are also expressed by macrophages and neutrophils and appear to play an important proinflammatory role in these cells. Muscarinic M_3_ and M_5_ receptors are expressed by macrophages, and muscarinic receptor agonists, such as carbachol, induce an increase in intracellular calcium and promote chemotaxis of these cells [60]. Alveolar macrophages also appear to express muscarinic M_1_, M_2_, and M_3_ receptor subtypes [13]. Stimulation by acetylcholine of these cells induces the release of leukotriene B_4_, which promotes neutrophil chemotaxis. This contention is in agreement with a study showing that, in bovine alveolar macrophages, muscarinic M_3_ receptors induce the release of leukotriene B_4_ [61]. Furthermore, it was recently shown that human alveolar macrophages respond to acetylcholine with the release of chemotactic activity for granulocytes, an effect likely involving leukotriene B_4_ release [47]. The anticholinergic agent tiotropium suppressed the secretion of leukotriene B_4_ by more than 70% after acetylcholine stimulation [47].

Treatment with tiotropium bromide significantly reduced airway inflammation and the Th2 cytokine production in bronchoalveolar lavage fluid (BALF) in both acute and chronic models of asthma. The levels of TGF-ß1 in BALF, the goblet cell metaplasia, thickness of airway smooth muscle, and airway fibrosis were all significantly decreased in tiotropium bromide-treated mice as well [62]. Tiotropium also concentration-dependently inhibited neutrophilic inflammation in response to cigarette smoke. Furthermore, the cigarette-smoke-induced pulmonary release of leukotriene B(4), interleukin-6, keratinocyte-derived chemokine, monocyte chemotactic protein-1, macrophage inflammatory protein-1 alpha and -2, and tumour necrosis factor alpha was dose-dependently reduced in murine model of COPD [63]. Neutrophil-elastase-induced goblet cell hyperplasia and gastrointestinal reflux-induced pulmonary inflammation can also be prevented by tiotropium treatment [64, 65]. These findings collectively indicate that acetylcholine, for example, nonneuronal acetylcholine derived from the inflammatory cells themselves, promotes inflammatory responses in the airways via muscarinic receptors.

## 4. The Nicotinic Receptor: Acetylcholine as an Anti-Inflammatory Mediator

The airway smooth muscle also expresses nicotinic receptors including the *α*3 and *α*7 nicotinic receptor subtypes [6]. The role of the nicotinic receptor in airway smooth muscle is currently largely unknown. However, in sharp contrast to the proinflammatory role of muscarinic receptor stimulation, nicotinic receptors appear to play an important anti-inflammatory role in many cell types and organs. Nicotinic receptors are found in the airways on parasympathetic nerves, macrophages, eosinophils, neutrophils, mast cells [66–70], lymphocytes [71–73], airway smooth muscle cells [74], epithelial cells [75], and fibroblasts [76].

Acetylcholine from neuronal or nonneuronal origin can induce an anti-inflammatory effect via *α*7 nicotinic receptors in various models of acute inflammation [77, 78]. This is also established in models of pulmonary inflammation including a mouse model of hypersensitivity pneumonitis [79], asthma [80, 81], and inflammation following influenza infection [82, 83]. These *in vivo* findings are supported by *in vitro* findings showing that stimulation of the *α*7 nicotinic receptor in murine macrophage cell lines results in inhibition of LPS-induced TNF and HMGB1 release [84–86]. Moreover, acetylcholine and nicotine receptor agonists exert a strong inhibitory effect on the release of TNF-*α* and other cytokines such as IL-6, IL-1*β*, IL-12, IL-18, and IFN-*γ* without affecting the production of anti-inflammatory cytokines although in some cases upregulation of IL-10 production is observed [79, 87–89]. Acetylcholine anti-inflammatory properties are regulated by *α*7 nicotinic receptor on macrophages, because macrophages from *α*7-subunit-nicotinic-receptor-deficient mice failed to show inhibition of TNF-*α* release [66]. Local administration of GTS-21 (a selective *α*7 cholinergic receptor agonist) also inhibits TNF-*α* release in the mouse lung during LPS-induced inflammation [90].

In addition to exerting anti-inflammatory effects on macrophages, activation of *α*7 nicotinic receptors on endothelial cells inhibits TNF-*α*-induced expression of intercellular adhesion molecule-1 and chemokines IL-8, RANTES (regulated on activation, normal T cell expressed and secreted), and monocyte chemoattractant protein-1 [91], thereby preventing migration of inflammatory cells from the blood to the tissues. Systemic administration of nicotine or a selective *α*7 agonist also attenuates acid-induced lung injury by reducing TNF-*α* and MIP-2 concentrations and by reducing neutrophil accumulation in the airspaces of the lung in rats, resulting in decreased pulmonary oedema and pulmonary inflammation [67]. Paradoxically, profibrotic, and proinflammatory effects of nicotine have also been reported, as nicotine appears to promote fibronectin deposition by fibroblasts [92]. Nonetheless, most reports point to an anti-inflammatory and antiremodelling role for the *α*7 nicotinic receptor. Thus, nicotinic agonists, including acetylcholine, can limit cytokine release and tissue inflammation. It was recently shown that *α*7 nicotinic receptors stimulation of alveolar macrophages and neutrophils also reduced chemokine production including MIP-2, transalveolar neutrophil migration, and LPS- and *E. coli*-induced acute lung injury in the airways of mice [67]. Collectively, these data indicate that acetylcholine may exert potent anti-inflammatory effects in the lungs, primarily via *α*7 nicotinic receptors. Although expressed by airway smooth muscle, the role of the *α*7 nicotinic receptor is currently unknown. Clearly, experiments to identify the role of the *α*7 nicotinic receptor in airway smooth muscle, including its role in remodelling and in the immunomodulatory function of airway smooth muscle, are warranted.

## 5. Conclusion

Acetylcholine is the predominant parasympathetic neurotransmitter in the airways and an autocrine or paracrine hormone. Many structural and inflammatory cells, notably the airway epithelium, express and secrete acetylcholine and respond to acetylcholine (either neuronal or nonneuronal) via muscarinic and nicotinic receptors. The airway smooth muscle is of major importance to the physiological and pathophysiological actions of acetylcholine, which induces bronchoconstriction, airway smooth muscle thickening, and the modulation of cytokine and chemokine production by these cells (Figure 1). Additionally, muscarinic receptors regulate proinflammatory and remodelling responses of fibroblasts and airway epithelial cells and promote the release of leukotriene B_4_ and other chemotactic mediators from macrophages and epithelial cells, resulting in eosinophil and neutrophil chemotactic activity.

 In contrast to this proinflammatory role, nicotinic receptors expressed by inflammatory cells and structural cells exert potent anti-inflammatory effects, in which the *α*7 nicotinic receptor appears to play a central role. This receptor subtype can be targeted both by neuronal and nonneuronal acetylcholine and may present a useful therapeutic target for treatment. The role of the *α*7 nicotinic receptor in airway smooth muscle, and in airway remodelling in asthma and COPD, is currently largely unknown but clearly warrants future investigation. In addition, it is essential to design future studies to identify the (patho)physiological basis for the clear discrepancy between nicotinic and muscarinic receptor subtypes in the regulation of inflammation and remodelling.

Clearly, the airway cholinergic system holds excellent therapeutic potential. Muscarinic receptor antagonists, currently widely used as bronchodilators for the treatment of COPD, may have beneficial anti-inflammatory and antiremodelling effects. Although direct evidence for this assumption is lacking in asthma and COPD patients, treatment with the anticholinergic agent tiotropium reduces exacerbation frequency in COPD patients and reduces lung function decline in GOLD stage II COPD patients [93, 94]. These clinical findings are consistent with anti-inflammatory and remodelling effects of tiotropium, but proof for this hypothesis still needs to be obtained. In addition, the anti-inflammatory effects of the *α*7 nicotinic receptor suggest that agonists for this receptor subtype are a strategy worth pursuing for the treatment of asthma and COPD.
